# Evaluation of removal efficiency of capping materials used in pulp revascularization in vitro

**DOI:** 10.1186/s12903-023-03313-3

**Published:** 2023-09-06

**Authors:** Gözde Yildiz Cebeci, Merve Erkmen Almaz, Ekim Onur Orhan, Arzu Beklen

**Affiliations:** 1 Private Practice, Ankara, 06100 Turkey; 2 Department of Pediatric Dentistry, Kırıkkale University Faculty of Dentistry, 71450 Kırıkkale, Turkey; 3 Department of Endodontics, Faculty of Dentistry, Eskisehir Osmangazi University, Eskisehir, 26040 Turkey; 4 Department of Periodontology, Faculty of Dentistry, Eskisehir Osmangazi University, Eskisehir, 26040 Turkey; 5 Translational Immunology Research Program (TRIMM), Research Program Unit (RPU), University of Helsinki, Helsinki, Finland

**Keywords:** Pulp revascularization, Failure, BioDentine, ProRootMTA, RetroMTA, Micro-CT analysis

## Abstract

**Background:**

This study aimed to evaluate the removal efficiency of different capping materials used in pulp revascularization (PR) in a failure scenario.

**Methods:**

The apices of freshly extracted 30 maxillary incisors were cut to mimic the immature teeth; then, root canals were shaped up to #6 Peeso reamers. The regeneration steps of the American Association of Endodontists (AAE) were followed to simulate PR treatment in vitro. The canals were dressed with the Ciprofloxacin and Metronidazole medicament mixture for 2 weeks. Then capping material groups were created: BioDentine (BD), ProRootMTA (PMTA), and RetroMTA (RMTA) (*n* = 10). The sealed specimens were stored for 2 weeks at 37 ºC in phosphate-buffered saline then the samples were examined by micro-computed tomography (µ-CT) analysis. Set capping materials were retrieved using a specific cement removal kit by a single blind operator. The residue materials were examined again by µCT. Kruskal–Wallis and Mann–Whitney U tests sought the significance for residue volumes. One-way ANOVA and Tukey post hoc tests with the Bonferroni corrections sought significance for the duration (*p* = 0.05).

**Results:**

In the first examined µCT data, the mean (SD) capping material volumes of the PMTA, BD, and RMTA were 6.447 µm^3^ (1.086), 8.771 µm^3^ (0.491), and 8.114 µm^3^ (2.447), respectively.

In the last examined µCT data, the median (IQR) residual volumes of the PMTA, BD, and RMTA were 0.051 µm^3^ (0.1), 0.313 µm^3^ (0.5), and 0.124 µm^3^ (0.1), respectively. A significant difference was found between BD and PMTA in the residual volumes (*p* < 0.05).

The mean (SD) durations of the retrieving procedures of PMTA, BD, and RMTA were 19.83 min (2.34), 19.24 (3.60), and 22.04 (1.68), respectively (*p* = 0.063).

**Conclusions:**

Within the limitations of the presented study, it was concluded that the capping materials were largely removed from the root canals using a non-invasive approach. Nevertheless, this duration of the retrieving could be described as long.

## Background

Pulp necrosis in an immature tooth can not only have a low prognosis with traditional treatment techniques but also have risks for complications [1]. Banchs & Trope [2] have suggested a ‘biologically-based treatment approach, namely pulpal revitalization (PR), for the management of the non-vital tooth with open apices instead of traditional apexification or single visit apical plug treatments. In the Glossary of Endodontic Terms, PR defines biologically-based stepwise processes developed to physiologically substitute damaged tooth structures, including pulp-dentin complex and root structures [3].

In PR, an infected root canal with immature teeth is recommended to be copiously irrigated with the standard cleaning solution at a low concentration (e.g., 1.5–3% sodium hypochlorite, NaOCl). In this stage, the disinfection is continued with calcium hydroxide or a low concentration of triple antibiotic paste dressing, then temporarily sealed for 1–4 weeks. At the following visit, the antibiotic mixture used in paste form is removed, the canal surface is conditioned with chelating agents, and apical bleeding is induced into the canal. At the final stage, the blood clot (tissue scaffold) is capped with a hydraulic tricalcium silicate cement (hTCS) positioned at 3–4 mm thickness or at the cervical third of the root, and a coronal restoration is placed after the initial setting reaction of hTCS has been completed [4].

Mineral trioxide aggregate (ProRoot MTA, Dentsply Tulsa Dental Specialties, Johnson City, TN, USA) is the first endodontic bioactive repair cement [5–7]. Today, the updated guidelines demonstrate the benefits of fast-setting hTCS counterparts as a capping material in PR [8]. However, the fast-setting counterparts of hTCSs can present differences in their adhesive properties to dentin substrate due to their varying ingredients [9–13]. Over the past decade, an extensive amount of research has been conducted on the bond strength of hTCS to dentin, which has yielded sufficient data to suggest that there can be significant differences in adhesive properties between their counterparts [9–13]. The effect of fast-setting hTCS counterparts is still unclear on PR procedures. A recent study mentioned that further studies on the manipulation effects of hTCS are necessary [4]. BioDentine (Septodont, Saint-Maur-des-Fosses, Cedex, France), known as a dentine substitute, is a fast-setting hTCS representative [14]. BioDentine contains Tricalcium silicate, dicalcium silicate, calcium carbonate, zirconium oxide, calcium oxide, and iron oxide [15]. RetroMTA (BioMTA, BioMTA, Yuseong-gu, Daejon, Korea) is a relatively newer hTCS that contains calcium carbonate, silicon oxide, aluminum oxide, hydraulic calcium zirconia complex, and water [16]. Due to their fast initial setting times and the fact that they do not induce discoloration of the teeth, it has been suggested to use them in vital pulp therapies and PR for both hTCSs [17].

The rationale of the endodontic retreatment is to completely remove old root canal filling material to avoid decreasing disinfectant cleaning efficiency. Accordingly, one of the main requirements of a root canal filling material is that it can be removed when necessary [18]. If there are signs or symptoms of persistent infection, additional antimicrobial visits in the scope of PR or traditional treatment alternatives must be considered in this event of failure [19, 20]. The clinical failure rate of PR was reported as 25% in immature incisors in a recent study [21]. In this context, it is still unclear whether completely removing the capping materials will be possible if any complications occur in PR. Furthermore, practitioners’ awareness and knowledge about removing solid (completed final setting reaction) hTCS from the cervical third of the canal are essential to managing the worst-case scenario in PR failures. Boutsioukis et al. [22] reported that some drawbacks might originate from removing hTCS from the root canal. These facts and justifications lead to base on the research question of this study. To the authors’ knowledge, no study has evaluated this research question in the literature. Based on the relevant question and the gap in the literature, the investigation of the removal efficiency of hCSCs is fundamental. Thus, the authors considered that this evaluation was necessary, particularly comparing capping materials with different properties.

This study aimed to evaluate the removal efficiency of different capping materials used in PR in a failure scenario. The null hypothesis of this study was there were no differences in the quantity of hTCS residues on the canal surface after the recommended removal techniques were applied.

## Methods

### Ethical approval

This study was approved by the Non-Invasive Medical Research Ethical Committee of the Kırıkkale University, Kırıkkale, Turkey; under the reference number: 2020.01.03 (issue date: 08.01.2020). Thirty teeth were collected and obtained from patients undergoing an extraction treatment at the Department of Surgery, Faculty of Dentistry, Kırıkkale University, Kırıkkale, Turkey. The informed consent agreement was obtained by all patients undergoing an extraction treatment and consenting to the use of their teeth for research purposes. The authors confirm that all experiments were performed in full accordance with the regulations and guidelines of the “World Medical Association Declaration of Helsinki in 2013” [23].

### Teeth selection

Sample size calculations for the study were performed using G*Power (v3.1, Heinrich Heine Universität Düsseldorf, Düsseldorf, Germany). Using the effect size obtained from the reference [24] as 0.50 and the sample size was calculated as 30. All samples were divided into 3 main groups per type of hTCS (*n* = 10). Thirty upper anterior teeth were collected for the current study. The teeth were gently cleansed from any residual debris or soft tissues under running tap water with a sharp hand scaler. Selected teeth were examined under x25 magnification to exclude any cracked, fractured, or defective teeth. Then teeth were reserved at 4°C in a 0.1% thymol solution up to a 3-month maximum period after extraction with changing the solution once per week untill use.

### Mimicking PR treatment

To mimic the immature teeth, the apices of tooth samples were cut using a low-speed diamond disc under water cooling at room temperature. The mean tooth length was standardized to be 10 ± 0.1 mm. Following the traditional endodontic access preparation, each root canal was enlarged using Peeso reamer drills from sizes 1 to 6 (Perfect Medical Instruments Co., Shenzhen, China). Root canals were copiously irrigated with deionized water between the root canal shaping.

The ‘American Association of Endodontists Clinical Considerations for a Regenerative Procedure’ was followed in this study [4]. The PR management and hTCS placements were carried out by an expert (M.E.A.). Each canal was irrigated with 20 mL of 1.5% sodium hypochlorite (Promida, Eskişehir, Turkey) using a side-vent needle (NaviTip, Ultradent, South Jordan, UT, USA) for 5 min, and then irrigated with 20 mL of 17% EDTA (Promida, Eskişehir, Turkey) for 5 min. The canals were then dried using ISO #140 size paper points (DiaPaper, DiaDent Group Int, Burnaby, BC, Canada). The double antibiotic paste was mixed with ciprofloxacin (Cipro 250, Biofarma, Istanbul, Turkey) and metronidazole (Flagyl 500, Eczacıbasi, Istanbul, Turkey) at a 1:1 ratio and prepared in paste form with glycerine at a concentration of 5 mg/ml. Each root canal was dressed with the medication and temporarily sealed with the Teflon barrier and IRM material (Lot# 1906000439; Dentsply Sirona, Milford, USA). The treated tooth samples were stored in sealed containers filled with phosphate-buffered saline (PBS, Lot#311010193; Elabscience, Houston, Texas, USA) for 2 weeks at 37 ºC in an incubator (Nuve EN 025, Ankara, Turkey).

**Fig. 1:**
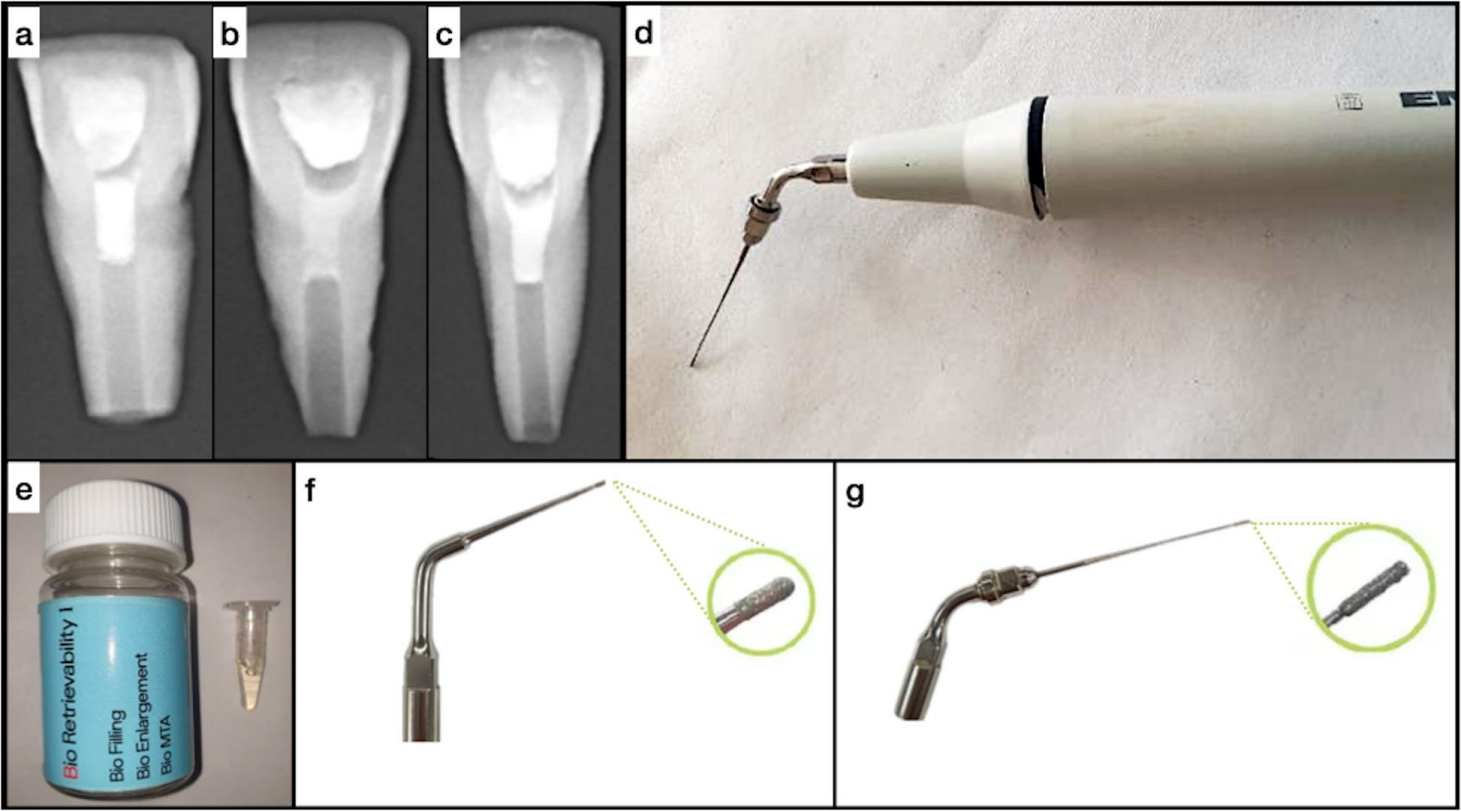
X-ray image data was acquired due to confirm the proper placement of (**a**) ProRoot MTA, (**b**) BioDentine, and (**c**) RetroMTA. **d** shows the Bust-05 tip and the attachment to the ultrasonic device. **e** shows the glycolic acid liquid used for softening capping material. **f** shows the magnification of the diamond-coated large ultrasonic tip (Bust-05). **g** shows the magnification of the diamond-coated fine ultrasonic tip (Bust-03)

Tooth samples were randomly divided into three capping material groups (*n* = 10): ProRoot MTA (PMTA, Lot#0000212745; Dentsply-Sirona, Tulsa, OK), BioDentine (BD, Lot#B24412; Septodont, Saint-Maur-des-Fosses, France), and RetroMTA (RMTA, Lot#1604D15; BioMTA, Yuseong-gu, Daejon, Korea). Groups of hTSCs were prepared specifically according to their manufacturer’s instructions. Each root canal was capped with hTSC at 3–4 mm thickness below the cementoenamel junction using a plugger. As soon as the initial setting time was achieved, the filled samples were stored again in the PBS for 2 weeks in the incubator Fig. 1. The information about the composition, initial setting time details, and mixing instructions of the capping materials is given in Table 1.

**Table 1:** The manufacturers’ disclosed information about the capping materials

**Capping material information**	**Powder compositions**	**Radiopacifier**	**Powder Color**	**Liquid ingredients**	**Setting type & durations**	**Mixing instruction**
**ProRootMTA** Lot# 0000212745 Expire date: 2022/09 Dentsply Sirona, Tulsa Dental Specialities, Johnson City, TN, USA	Tricalcium silicate, Dicalcium silicate, Tricalcium aluminate, Tetracalcium aluminoferrite, Free calcium oxide, Bismuth oxide	Bismuth oxide	White	ProRoot liquid in micro-dose ampoule	*Slow-setting* Initial: 74 min Final: 165 min	0.5 g pouches of powder + pre-measured unit dose of water at 3:1 powder-to-liquid ratio (mixed manually)
**RetroMTA** Lot# 1604D15 Expire date: 2022/07 BioMTA, Yuseong-gu, Daejon, Korea	Calcium carbonate, silicon oxide, hydraulic calcium zirconia complex, aluminum oxide	Zirconium oxide	White	RetroMTA liquid in micro-dose ampoule	*Flash-setting* Initial: 150‐180 s Final: 360 min	The 0.3 g powder pours onto the three drops of its liquid and wets it gently for 20 s. when the shiny surface disappears, the gel-form is applied (mixed manually)
**BioDentine** Lot#: B24412 Expire date: 2022/09 Septodont, Saint-Maur-des-Fosses, Cedex, France	Tricalcium silicate, dicalcium silicate, calcium carbonate, zirconium oxide, calcium oxide, iron oxide	Zirconium oxide	White	0.2 g water-based calcium chloride as the setting accelerator in micro-dose ampoule	*Flash-setting* Initial: 9-12 min Final: 45 min	5 drops of liquid pours into the 0.7 g capsule of powder, then mixed for 30 s at 4000-rpm

### Retrieving capping materials and data collection

The data used for this study were collected by micro-computed tomography (µCT) analysis. For this purpose, a Skyscan 1272 µCT instrument (Bruker Corp., Billerica, MA, USA) was used in the scans. The samples were fixed on the turntable of the µCT instrument, and the X-ray source was irradiated at 90 kV, 100 mA beam current, 0.5 mm Al/Cu filter rate, 33 μm pixel size, and 0.5 rotation steps. Each sample was rotated 360° within an integration time of 5 min, and the total scan duration was set at 45 min for each sample. Other settings and parameters of the instrument were made in accordance with the manufacturer’s recommendations. The quantification of the hTCS was examined in the CTVox software (v2.2.3; Bruker Corp., Billerica, MA, USA).

The single-blind operator (G.Y.C.) performed retrieval procedures for capping materials in vitro. The 2-week-old capping materials were retrieved using an ultrasonic hTCS removal kit (BioMTA, Yuseong-gu, Daejon, Korea). In the retrieval approach, the manufacturer’s instructions were strictly followed for each step. The procedure was summarized as follows:

1. The coronal surface of the material was softened with a single drop of 10% glycolic acid (Lot#BR-1/180910; Bio Retrievability I; BioMTA, Yuseong-gu, Daejeon, Korea) for 5 min.
2. Ultrasonic tips of ‘Bust-05’ (Lot#151014–001, BioMTA) and ‘Bust-03’ (Lot# A16L000300, BioMTA) were gently operated under 3.5X magnification (ProMag, Carl Zeiss, Germany), respectively, at the lowest output of an ultrasonic instrument (E.M.S. Electro Medical Systems S.A., Nyon, Switzerland) until capping material was completely eliminated. Each canal was copiously irrigated with saline during the procedure and dried with paper points Fig. 1.

**Fig. 2:**
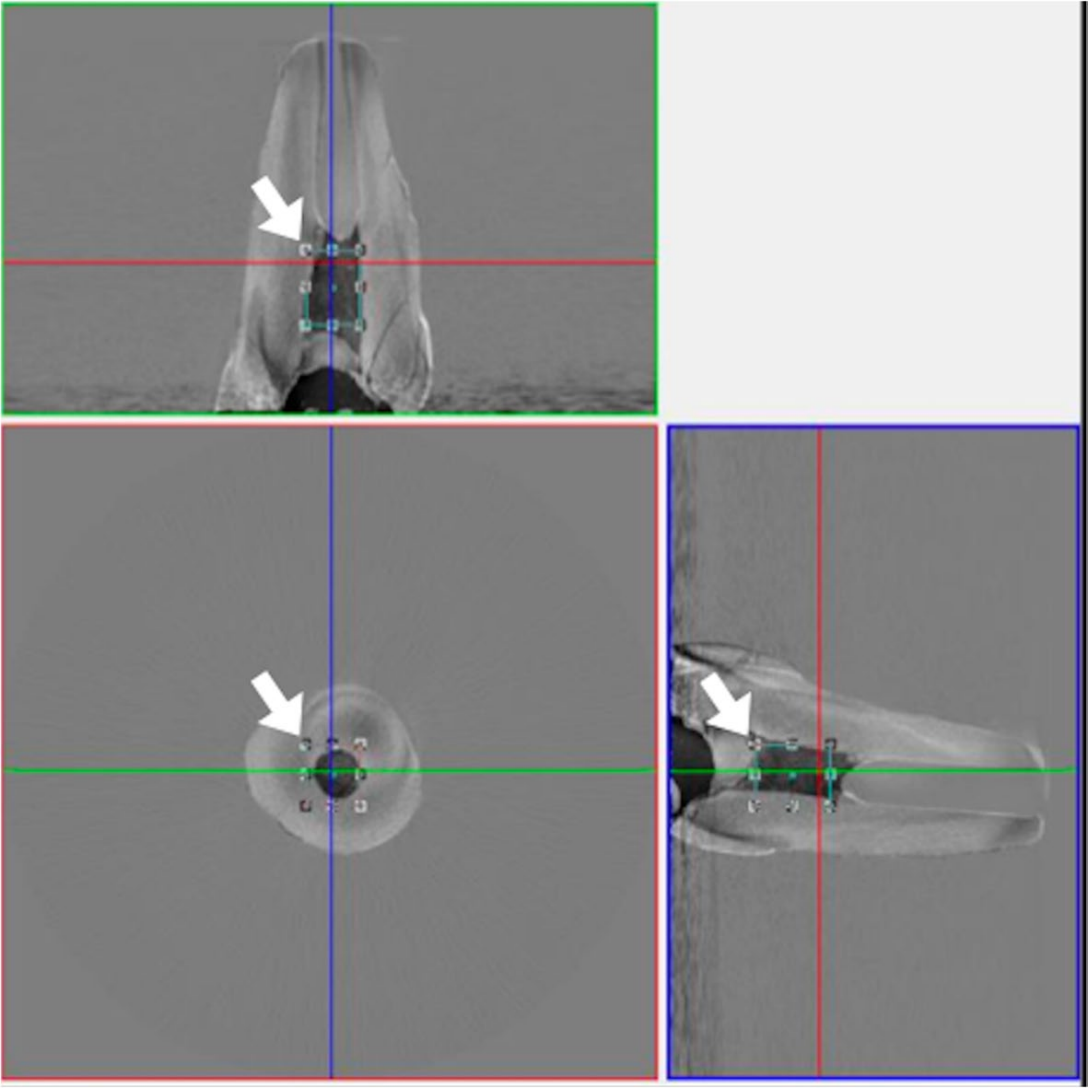
The representative examination of µ-CT data (left) shows the determination of the regions of interest at different planes (white arrows). The representative screenshot of CTAn software (right) shows an image analysis of the overlapped scans (red arrow)

Following the retrieval procedures, all samples were placed in the holder of the µCT instrument according to the groups and scanned a second time under the same conditions using the parameters specified in the first scan. The quantification of the hTCS residue was examined in the CTVox software Durations for each procedure were recorded by an observer (M.E.A.) using the chronometer application of an IOS smartphone Fig. 2.

### Statistical analysis

The normality of the data was tested using the Shapiro-Wilks test. Statistical analyses were performed using SPSS (v21; IBM Corp., Armonk, NY). Kruskal–Wallis and Mann–Whitney U tests sought the significance of residue volumes. One-way ANOVA and Tukey post hoc tests with Bonferroni corrections sought significance for the duration. The significance level was set at 5%.

## Results

The dataset is available in the citation [25]. The analyzed data for µCT volumes and durations are given in Table 2.

**Table 2:** The analyzed data presentation of µCT examinations and durations

**Analysis**	**Groups**	**n**	**Median**	**min**-**max**	**IQR**	**Mean**	**SD**	***p***
**I. µCT examination data** Initial volume (µm^3^)	ProRoot MTA		-	4.601-7.764	-	6.447	1.086	
	BioDentine	10	-	7.867-9.388	-	8.771	0.491	
	RetroMTA		-	4.180-12.269	-	8.114	2.447	
**II. µCT examination data** Residual volume (µm^3^)	ProRoot MTA		0.051^a^	0.001-0.303	0.1	-	-	< .05^^^
	BioDentine	10	0.313^bc^	0.015-0.759	0.5	-	-	
	RetroMTA		0.124^ac^	0.004-0.328	0.1	-	-	
**Durations of the retrieving procedures (min)**	ProRoot MTA	10	-	17.55-24.28	-	19.83	2.34	
	BioDentine		-	12.67-23.71	-	19.24	3.60	0.063^*^
	RetroMTA		-	20.08-25.05	-	22.04	1.68	

*IQR* Interquartile range, *SD* Standard deviation

^^^Mann–Whitney U test *p* value

^*^Post-hoc with the Bonferroni corrections test *p* value. The same superscript lowercase shows no significance

In the first examined µCT data, the mean (SD) capping material volumes of the PMTA, BD, and RMTA were 6.447 µm^3^ (1.086), 8.771 µm^3^ (0.491), and 8.114 µm^3^ (2.447), respectively.

**Fig. 3:**
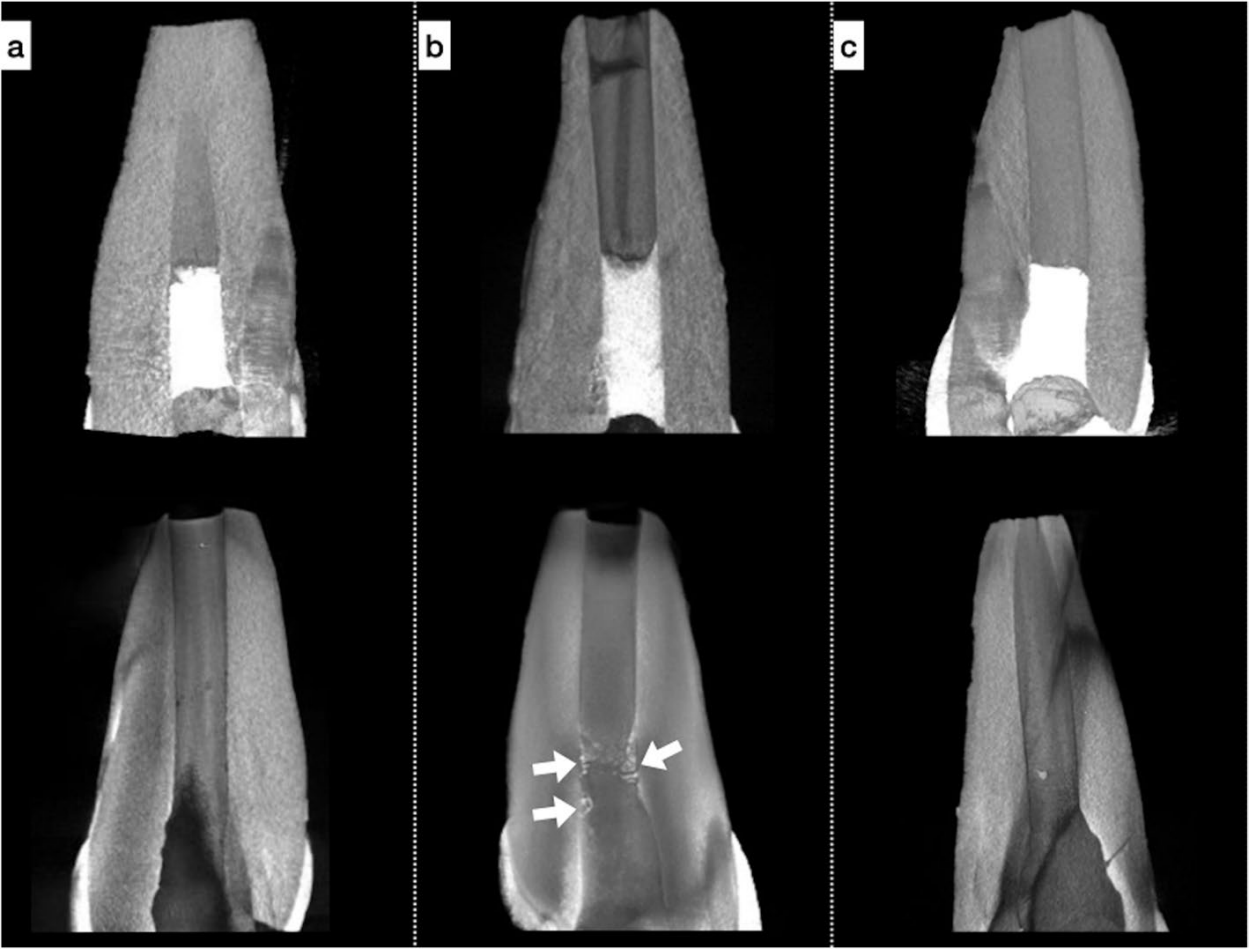
The representative examination of µCT data of (**a**) ProRoot MTA, **b** BioDentine, and (**c**) RetroMTA. Upper image data show the initial examination, whereas the last examinations are shown below. The white arrow shows residual material in the BioDentine

In the last examined µCT data, the median (IQR) residual volumes of the PMTA, BD, and RMTA were 0.051 µm^3^ (0.1), 0.313 µm^3^ (0.5), and 0.124 µm^3^ (0.1), respectively Fig. 3. A significant difference was found between BD and PMTA in the residual volumes (*p* < 0.05).

The mean (SD) durations of the retrieving procedures of PMTA, BD, and RMTA were 19.83 min (2.34), 19.24 (3.60), and 22.04 (1.68), respectively (*p* = 0.063).

## Discussion

This study evaluated the removal efficiency of different capping materials used in PR in a failure scenario. To achieve this aim, for the first time, research was conducted on a method of hTCS removal that was considerably less invasive. High-speed rotary devices could be used to remove hTCSs; however, because immature teeth have thin root dentin, this could pose a threat to the teeth’s ability to survive. The white-colored capping materials present low contrast to the dentine substrate. This may lead to difficulties in the optical discrimination of small residues by operators. The highest median value of residue material was seen in the BD, significantly different from PMTA. Therefore, the null hypothesis was rejected.

A single operator experienced in manipulating hTCS materials placed the capping material in the PD simulation. Although the root canal spaces of tooth samples were standardized, the volume of the capping materials was seen to vary in the first µCT examination. This could be originate from the differences in their handling characteristics of hTCSs [8].

The incisor teeth were preferred in the PR simulation due to being frequently affected by dental trauma in school-age children and therefore having a risk of losing vitality [26]. The authors noted that the 2-week-old capping material needs 20–25 min of chair time for the removal procedures in clinical practice. Considering school-age children, this duration could be described as long.

In PR guidelines, hTCS cement has been suggested as a capping material [1, 4]. To undertake biologically-based healing, the prominent rationale for the current suggestion is based on the good bioactivity, biocompatibility, and adhesion properties of hTCSs [5–8, 27]. In our findings, the differences in the amount of residual capping material were attributed to the differences in adhesive properties between PMTA, BD, and RMTA. Thus, literature-based adhesive data for PMTA, BD, and RMTA, and their physical characteristics and ingredient details affecting adhesion were discussed in this part.

The precursor hTCS counterpart, PMTA, has a relatively slow initial setting time (74 min) and has been reported to cause tooth discoloration [28]. On the contrary, its successors, RMTA or BD, have a flash-initial setting time and do not cause discoloration [28]. More specifically, the initial setting time of RMTA and BD has been reported as 3 min [16] and 12 min [15], respectively. Hench reported that the flash initial setting reactions of the bioceramics could induce more mineral attachment on the surface of the dentin substrate [29]. Comparative in vitro studies reported that BD had a higher bond strength than PMTA to dentin [30–33]. The shear bond strengths of 2-week-old BD and PMTA were reported as 9.34 ± 1.01 MPa and 4.96 ± 4.54 MPa in a previous study (*p* < 0.05) [30]. Likely, the push-out bond strength of 7-day-old PMTA was reported as 4.75 ± 1.71 MPa and 9.0 ± 0.9 MPa in previous studies [31, 32].

Furthermore, the previously reported relatively smaller granular size and uniform powder distribution of BD might contribute to the micromechanical anchoring to the dentine since the cement characteristics affect the marginal adaptation of hydrated cement materials [13, 34]. Moreover, different failure modes were reported between BD and PMTA in a previous study [30]. Accordingly, it has been reported that BD has a more cohesive type failure mode; whereas, PMTA has a more adhesive type failure mode [30].

It has been reported that the presence of residual irrigation solutions, antibiotics or calcium hydroxide medicaments, and the smear layer affect the bond strength of hTCS [11, 13]. The simulation of the capping material placement and storage did not fully mimic the clinical conditions due to the experiment’s absence of blood plasma or blood clot products. This could be considered a limitation of the study.

When PMTA was first introduced, it was not considered for retrieval after hTCS administration as it was a retrograde filling and repair material [35]. However, in the case of contemporary non-invasive treatment failures, the set hTCS material must be completely removed to allow conventional treatments to be performed. Old canal filling residues affect the subsequent sealing properties, especially the tubule penetration depth [36]. Following the conservative technique utilized, the evaluation suggests that hTCS removal is most likely to be complete with an invasive approach such as Gates Glidden rotary instruments. However, invasive methods would have risks of complication due to the weakness of root dentin in immature teeth. In addition, the authors suggested that removing hTCS after failed regenerative endodontic procedures could be a problem. Further studies are needed to better understand the effect of hTCS removal on the outcome of further treatment.

## Conclusions

Within the limitations of the presented study, it was concluded that the capping materials were largely removed from the root canals using a non-invasive approach. Nevertheless, this duration of the retrieving could be described as long.

## Data Availability

The datasets used and/or analysed during the current study available from GY Cebeci gozdde.yildizz@gmail.com on reasonable request. The dataset is available in the citation [25].

## Abbreviations

µCT: Micro-computed tomography
AAE: American Association of Endodontists (AAE)
BD: BioDentine
hTCS: Hydraulic tricalcium silicate cement
PBS: Phosphate-buffered saline
PMTA: ProRootMTA
PR: Pulp revascularization
RMTA: RetroMTA
SD: Standard deviation
